# Severe cellular stress drives apoptosis through a dual control mechanism independently of p53

**DOI:** 10.1038/s41420-022-01078-2

**Published:** 2022-06-09

**Authors:** Yen-Chun Wang, Li-Ting Wang, Ta I Hung, Yi-Ren Hong, Chung-Hwan Chen, Cheng-Jung Ho, Chihuei Wang

**Affiliations:** 1 Department of Medical Education, Taipei Veterans General Hospital, Taipei, 11217 Taiwan; 2 Department of Biotechnology, Kaohsiung Medical University, Kaohsiung, 80708 Taiwan; 3 Department of Bioengineering, University of California, Riverside, Riverside, CA 92521-0429 USA; 4 Department of Biochemistry & Graduate Institute of Medicine, Kaohsiung Medical University, Kaohsiung, 80708 Taiwan; 5 Department of Orthopedics, Kaohsiung Medical University Hospital, Kaohsiung, 80708 Taiwan; 6 Graduate Institute of Clinical Medicine, Kaohsiung Medical University, Kaohsiung, 80708 Taiwan

**Keywords:** Cancer therapy, Cell death

## Abstract

For past two decades, p53 has been claimed as the primary sensor initiating apoptosis. Under severe cellular stress, p53 transcriptional activity activates BH3-only proteins such as Bim, Puma, or Noxa to nullify the inhibitory effects of anti-apoptotic proteins on pro-apoptotic proteins for mitochondrial outer membrane permeabilization. Cellular stress determines the expression level of p53, and the amount of p53 corresponds to the magnitude of apoptosis. However, our studies indicated that Bim and Puma are not the target genes of p53 in three cancer models, prostate cancer, glioblastoma, and osteosarcoma. Bim counteracted with Bcl-xl to activate apoptosis independently of p53 in response to doxorubicin-induced severe DNA damage in prostate cancer. Moreover, the transcriptional activity of p53 was more related to cell cycle arrest other than apoptosis for responding to DNA damage stress generated by doxorubicin in prostate cancer and glioblastoma. A proteasome inhibitor that causes protein turnover dysfunction, bortezomib, produced apoptosis in a p53-independent manner in glioblastoma and osteosarcoma. p53 in terms of both protein level and nuclear localization in combining doxorubicin with bortezomib treatment was obviously lower than when using DOX alone, inversely correlated with the magnitude of apoptosis in glioblastoma. Using a BH3-mimetic, ABT-263, to treat doxorubicin-sensitive *p53*-wild type and doxorubicin-resistant *p53*-null osteosarcoma cells demonstrated only limited apoptotic response. The combination of doxorubicin or bortezomib with ABT-263 generated a synergistic outcome of apoptosis in both *p53*-wild type and *p53*-null osteosarcoma cells. Together, this suggested that p53 might have no role in doxorubicin-induced apoptosis in prostate cancer, glioblastoma and osteosarcoma. The effects of ABT-263 in single and combination treatment of osteosarcoma or prostate cancer indicated a dual control to regulate apoptosis in response to severe cellular stress. Whether our findings only apply in these three types of cancers or extend to other cancer types remains to be explored.

## Introduction

Regulated cell deaths (RCDs) are classified into eleven categories so far, including apoptosis and ten non-apoptotic types of cell death [1]. Apoptosis represents the tightly controlled process that removes not only damaged cells but also superfluous cells during tissue differentiation and renewal [2, 3]. Signals from the external environment or internal cellular stress control this main RCD. The respective action induced by outside or inside signals is regarded as extrinsic or intrinsic apoptosis [4].

Intrinsic apoptosis occurs in response to intracellular stresses like DNA damage or endoplasmic reticulum stress. Although the specific signal pathway might be activated corresponding to the type of cellular stress, the intrinsic apoptosis pathway eventually proceeds to mitochondrial outer membrane permeabilization (MOMP), which is regulated by the Bcl2 family [5, 6]. So far, about 25 members of the Bcl2 family have been identified [7]. They constitute three subgroups, including anti-apoptotic, pro-apoptotic, and BH3-only proteins. Two alternative models, direct activator–derepressor (DAD) and indirect activator (IA), have been proposed to depict how BH3-only proteins counteract anti-apoptotic proteins to activate pro-apoptotic proteins for MOMP [6]. The DAD claims that BH3-only proteins consist of two subgroups, Bad, BMF, Noxa, Bik, or HRK working as sensitizers, and Bim, Bid, or Puma functioning as activators. In the absence of cellular stress, the activators are bound by the anti-apoptotic proteins, Bcl2, Bcl-xl, or Mcl-1, to remain in an un-activated condition. Under cellular stress, the sensitizers will displace the activators from the anti-apoptotic proteins. The free activators activate Bax/Bak to form oligomer pores for MOMP. In contrast, the BH3-only proteins dislodge Bax/Bak from the anti-apoptotic protein complex to form oligomers for MOMP via the IA model. MOMP causes the release of cytochrome *c* from the mitochondria into the cytoplasm, resulting in the formation of the apoptosome and caspase 3 or 7 activation [8]. The activated caspase 3 and 7 are the executors of apoptosis [9].

The controllers of extrinsic apoptosis are death receptors, including Fas, the tumor necrosis factor (TNF) receptors TNFR1 and TNFR2, and the TNF-related apoptosis-inducing ligand (TRAIL) receptors DR4 and DR5 [10, 11]. By binding to the corresponding ligand, death receptors acquire adapter proteins such as FADD, and initiator caspases such as caspase 8 and caspase 10, resulting in the activation of executed caspases like caspase 3 and caspase 7, and the activation of a BH3-only protein, Bid. Bid can enhance apoptosis through MOMP.

The chemotherapeutic agents that produce severe cellular stress to trigger apoptosis mainly function through intrinsic apoptosis [12]. The tumor suppressor gene, p53, is regarded as an important sensor in this pathway [13, 14]. The first evidence from a study of p53 in apoptosis demonstrated that overexpression of temperature-sensitive mutant p53 can drive cells into death at permissive temperatures [15]. Then Puma and Noxa, which are BH3-only proteins responsible for apoptosis, were identified as p53 target genes [16, 17]. Their expression always accompanies p53 when SDD is induced by chemical agents or radiation. Interestingly, mutated or missing p53 causes resistance to chemotherapy in many human cancers [18]. To overcome such resistance, researchers developed new BH3-mimetic therapeutic agents that can bind to anti-apoptotic Bcl2 members, inhibit their function and directly activate the apoptotic pathway, bypassing the p53-mediated pathway [19, 20].

Here, we showed that p53 has no function in SDD- and PTD-induced apoptosis, at least in prostate cancer, glioblastoma, and osteosarcoma. Moreover, by combining a BH3-mimetic with other therapeutic agents to treat prostate cancer and osteosarcoma, we clarified the mechanism by which severe cellular stress induces apoptosis through the dual control pathway.

## The function of p53 in SDD- or PTD-induced apoptosis

Cancer chemotherapy to produce severe cellular stress mainly act by the induction of intrinsic apoptotic pathways [12]. Agents that cause DNA damage and disruption of protein turnover have been applied in chemotherapy for various types of cancers. Doxorubicin (DOX) and bortezomib (BTZ), which correspond to SDD and PTD to drive cancer cells into apoptosis.

DOX functions by topoisomerase II poisoning [21] and also directly intercalates into DNA [22]. It causes a DNA damage response (DDR), resulting in apoptosis. p53 functions as an executor in DDR, as a molecular switch between cell cycle arrest and apoptosis depending on the level of DNA damage [13]. The intensity of DNA damage determines the expression of p53, and therefore the threshold of molecular switching is the amount of p53 [23]. Below the threshold, p53 activates the genes responsible for cell cycle arrest and DNA repair including *p21, GADD45A, DDB2, TANCC*, and *XPC* [24, 25]. In contrast, the genes participating in apoptosis, such as *Noxa, Puma, Bim,* and *Bax*, will be activated transcriptionally by p53 in conditions above threshold values [13, 24].

Targeting the ubiquitin-proteasome pathway (UPP) shows great potential for cancer therapy [26]. Regarding UPP inhibitors, the most representative agent is BTZ, which has been proven to treat multiple myeloma and mantle cell lymphoma [27, 28]. BTZ specifically binds to 20S catalytic core of 26S proteasome to block UPP, engendering PTD stress [29]. The impairment of NF-κB, the stabilization of p53, pro-apoptotic proteins or BH3-only proteins, the depletion of ubiquitin, the increase of ER stress, or JNK pathway action due to PTD stress are all possible reasons for BTZ-induced apoptosis [30, 31]. There are studies to claim BTZ-induced apoptosis through p53-dependent pathway [32, 33] or p53-independent pathway [34, 35].

## SDD-induced apoptosis in prostate cancer

Two prostate cancer cell lines, *p53*-wild-type (WT) LNCaP and *p53*-null PC3, were used to address p53 function in SDD-induced apoptosis [36]. DOX could induce apoptosis in both LNCaP and PC3 cells. DOX drove LNCaP cells toward the maximization of apoptosis, but it merely initiated apoptosis in PC3 cells and halted even with increasing concentrations of DOX. DOX was capable of initiating cell apoptosis with/without p53, but it was only able to maximize apoptosis in cells with p53. The BH3-only proteins, Bim and Puma, were seemingly not the target genes of p53, since both were about equally expressed in LNCaP and PC3 cells. Moreover, Bim was responsible for apoptosis induced by SDD. To define acting mechanism of Bim, the BH3-mimetic, ABT-263, in single agent or in combination with DOX were used to treat LNCaP and PC3 cells. ABT-263 alone could generate apoptosis in both cells with low efficacy, while apoptotic response was higher in the combination of DOX with ABT-263 than that in using DOX or ABT-263 alone. Bim knockdown did not affect apoptosis induced by ABT-263, suggested that Bim might just like ABT-263 that worked as a sensitizer to counteract Bcl-xl. Since the expression of Bim was about equally between p53-WT LNCaP and p53-null PC3, p53 likely played no role to activate this sensitizer by counteracting Bcl-xl in response to DOX-induced SDD [36].

Overexpression of p53 had no effect on apoptosis induced by ABT-263 and the synergistic effect of ABT-263 on DOX-induced apoptosis only appeared in the high concentration of DOX that causes SDD [37]. Overexpression of p53 enhancing apoptosis also occurred in DOX-induced SDD, not in DOX-induced cell cycle arrest [37]. Thus, we speculated that the determined factor of apoptosis was the level of cellular stress, not the amount of p53. We asked if p53 activated transcriptionally unknown factors to couple with Bim for apoptosis in response to SDD.

**Fig. 1:**
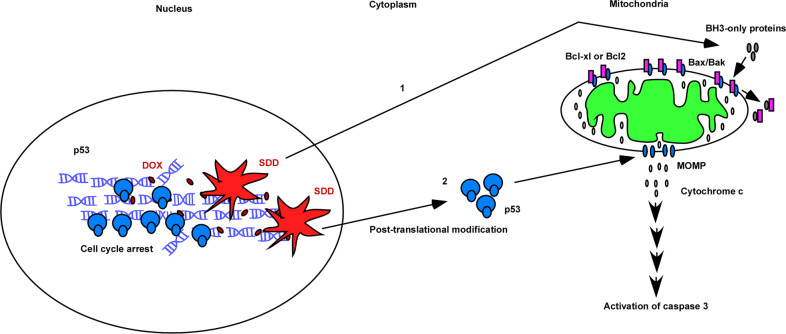
SDD activated BH3-only proteins (Bim) and p53 proteins to work as sensitizers and activators, respectively, through the dual control mode for MOMP. Bim action was in p53-independent manner and the transcriptional activity of p53 did not involve in SDD-induced apoptosis. Cell death increased in over-expressed p53 condition and the substantial amounts of p53 localized in cytosol during apoptosis in prostate cancer. Based on these results, we initially proposed that p53 proteins in cytosol might work as activators in SDD-induced apoptosis. However, the role of p53 proteins as activators was not supported by the results from the studies of glioblastoma and osteosarcoma. Thus, we thought that cell death caused by p53 overexpression condition might not account for the real physiological role of p53 in apoptosis and p53 might not be an activator in the dual control model.

Significantly, the transcriptional activity of p53 was not involved in DOX-induced apoptosis in LNCaP cells [38]. We thought that the transcription-independent activity of p53 might involve in SDD-induced apoptosis. The BH3-only protein, Bim, worked as a sensitizer to release Bax/Bak from Bcl-xl and p53 worked as an activator to activate Bax/Bak in response to SDD, named as the dual control pathway for MOMP (Fig. 1).

## p53-independent SDD- and PTD-induced apoptosis in glioblastoma

Two cell lines of glioblastoma, *p53-*WT U87 and *p53*-mutation T98G cells, were assessed the apoptotic response to DOX and BTZ [39]. DDR caused by DOX could be characterized into two events: p21 expression for cell cycle arrest and caspase 3 activation for apoptosis, corresponding to mild and severe DNA damage conditions in U87 cells. When heavy apoptosis occurred at 1 μM of DOX, p53 mostly concentrated into the nucleus. Interestingly, the transcriptional activity of p53 also had no effect on apoptosis in glioblastoma. It was almost impossible for p53 in the nucleus to perform transcription-independent apoptosis, challenging the transcription-independent activity of p53 in the dual control of apoptosis (Fig. 1).

PTD induced by BTZ could efficiently activate apoptosis in U87 and T98G, indicating that BTZ’s effect on apoptosis was p53-independent. The combination of BTZ with DOX showed a much higher apoptotic response than either BTZ or DOX alone in U87. In other words, the compound stress generated by SDD plus PTD synergized apoptosis in U87. Interestingly, p53 in terms of both protein level and nuclear localization in combination treatment was obviously lower than when using DOX alone. The concentration of p53 in the nucleus was correlated with DNA damage other than apoptosis. Translocation of p53 into the nucleus might be for cell cycle arrest in DDR rather than being related to apoptosis.

## p53-independent SDD- and PTD-induced apoptosis in osteosarcoma

**Fig. 2:**
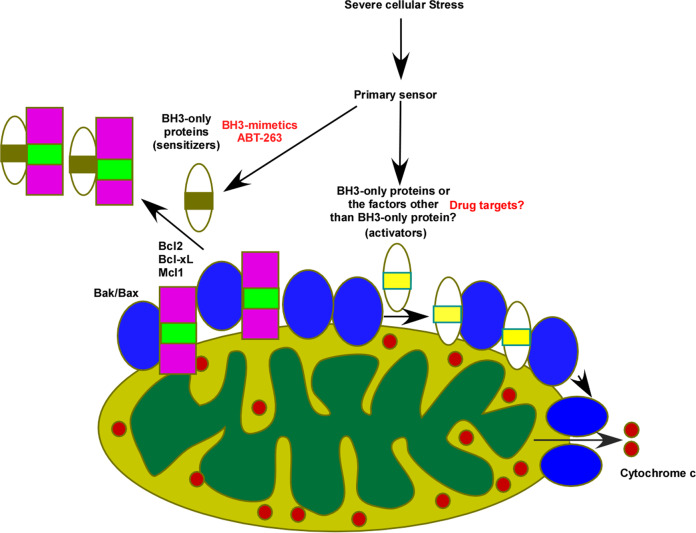
The dual control model for MOMP. In response to severe cellular stress, sensitizers might disentangle Bax/Bak from anti-apoptotic proteins such as Bcl2 or Bcl-xl and then activators might prompt the formation of Bax/Bak pore for MOMP in p53-independent manner. These sensitizers might be BH3-only proteins, nevertheless these activators might be also BH3-only proteins or the factors other than BH3-only protein.

Two osteosarcoma cell lines, *p53*-WT U2OS and *p53*-null MG63 cells, were used to analyze the apoptotic response to SDD and PTD [40]. Neither Bim nor Puma were target genes of p53. The activation of apoptosis appeared slightly in response to DOX-induced SDD at 2 μM and increased significantly at 4 μM in U2OS cells. Even with DOX up to 4 μM, apoptosis could not be activated in MG63 cells, indicating that the BH3-only protein responsible for initiating apoptosis might be lost in this cell. While ABT-263 alone could induce a low degree of apoptosis in U2OS and MG63, the combination of DOX with ABT-263 demonstrated a synergistic effect on apoptosis with about same efficiency in both cells. ABT-263 that might function as a sensitizer released pro-apoptotic proteins from Bcl2 or Bcl-xl, and then SDD induced by DOX stimulated activators to promote pro-apoptotic proteins for MOMP. Since the osteosarcoma cells with/without p53 had same response to DOX plus ABT-263, we concluded that p53 had no role in SDD-induced apoptosis. Thus, the dual control model (Fig. 1) was refined into the rational route excluding the role of p53 in apoptosis (Fig. 2).

Different from DAD model, we claimed that severe cellular stress affected sensitizers and activators in two separated pathways (Fig. 2). The synergistic apoptosis induced by the combination of DOX with ABT-263 appeared in LNCaP, U2OS, and MG63, not in PC3 [40]. DOX could slightly induce apoptosis in PC3 [36] whereas it almost had no effect on MG63 [40]. Thus, we speculated that LNCaP or U2OS might contain both sensitizers and activators, nevertheless PC3 or MG63 might own sensitizers or activators only, respectively. Clearly, sensitizers and activators might possess their own distinguish functions, other than that distinctions between sensitizers and activators are not absolute [41]. Moreover, the mouse fibroblasts lacking all of Bim, Bid, Puma, and Noxa still sustain apoptosis in response to DNA damage [42], suggested that other forms of activators might exist [41]. Therefore, we thought that the sensitizers were BH3-only proteins, nevertheless the activators could be also BH3-only proteins or the factors besides BH3-only proteins (Fig. 2).

BTZ efficiently triggered apoptosis in both U2OS and MG63 cells, suggesting that PTD-induced apoptosis was p53-independent in osteosarcoma. The synergistic effect on apoptosis induced by the combination of BTZ with ABT-263 suggested that a specific BH3-only protein might serve as a sensitizer signal to couple with an activator signal for PTD-induced apoptosis, similar with SDD-induced apoptosis (Fig. 2).

## Discussion and conclusions

Studies in prostate cancer, glioblastoma, and osteosarcoma demonstrated that p53 might have no function in SDD-induced apoptosis. Particularly, in osteosarcoma direct evidence showed that SDD-induced apoptosis was p53-independent. Moreover, p53 also did not participate in PTD-induced apoptosis in glioblastoma and osteosarcoma. By combining ABT-263 with DOX or BTZ, we established the dual control model for SDD- and PTD-induced apoptosis (Fig. 2).

The evidences that p53 has the capacity for apoptosis comes from experiments overexpressing a temperature-sensitive p53 at permissive temperatures, or WT p53 in inducible or transient transfection systems in various cancer cells [15, 23, 37]. Furthermore, the BH3-only proteins such as Bim, Puma, and Noxa that are essential regulators of apoptosis have been identified as p53 target genes [14]. Based on the above findings, the model of a p53-mediated apoptotic pathway in response to severe cellular stress, particularly SDD, has been built up. Since cancer cells with missing or mutated p53 consistently display chemotherapy-resistant phenotypes, the function of p53 in apoptosis has been conceded for the past three decades. It is suggested that the cancer cells, which lost transcriptional function of p53, might not activate Bim, Puma, or Noxa to trigger apoptosis for responding to chemotherapy agents [43]. Bim and Puma are activators for Bax/Bak and Noxa functions as the sensitizer to inhibit anti-apoptotic proteins. Recently, Noxa or Bim is also recognized as an activator [42] or a sensitizer [36], respectively. Whether the segregation of BH3-only proteins into activators and sensitizers is just for convenience [41] or for their distinctive functions became interesting.

p53 is a powerful transcription factor responsible for nearly 500 gene expressions [24]. The target genes of p53 deal with many cellular functions including cell cycle arrest, cell senescence, DNA repair, metabolic adaptation and cell death. Enforced expression of p53 might generate cellular stress by the accumulation of unnecessary proteins of p53 target genes, causing cell death. This effect might not represent the real physiological role of p53 in apoptosis. Moreover, the BH3-only proteins Bim and Puma were constitutively expressed in prostate cancers, glioblastoma and osteosarcoma cells with or without functional p53 [36, 39, 40]. The reports have also demonstrated that embryonic stem cells undergo p53-independent apoptosis in response to DNA damage [44] and p53-null osteosarcoma Saos-2 cells are induced toward p53-independent apoptosis by DOX [45].

The main function of p53 is to protect the integrity of the genome from DNA damage. Without p53 protection, other genes are easily to loss or mutate. Cancer cells without functional p53 might generate chemotherapy resistance because of the missing or mutation of apoptotic regulators, such as Bim, Puma, Noxa or others. Based on the dual control model (Fig. 2), severe cellular stress-induced different pathways to regulate sensitizers and activators, and they needed to be clearly distinguished functionally. In addition, there might be other apoptotic regulators, notably activators, still uncharacterized. A more complete dissection of apoptotic pathways will provide more molecular markers to verify the efficacy and strategy of chemotherapy.

Moreover, targeting the apoptotic pathway has become a convincing goal of drug discovery, mainly due to the successful development of BH3-mimetics [46]. Unfortunately, one-sided inhibition by BH3-mimetics cannot effectively activate apoptosis without severe cellular stress. Based on the dual control model, the BH3-mimetic that interacts with Bcl2 or Bcl-xl would couple with drugs designed for the second as-yet-uncharacterized apoptotic control, maximizing apoptosis without severe cellular stress.

## Data Availability

The datasets used and/or analyzed during the current study are available from the corresponding author on reasonable request.
